# A Recombinant Chimera Vaccine Composed of LTB and *Mycoplasma hyopneumoniae* Antigens P97R1, mhp390 and P46 Elicits Cellular Immunologic Response in Mice

**DOI:** 10.3390/vaccines11081291

**Published:** 2023-07-28

**Authors:** Wei Liu, Peizhao Jiang, Tao Song, Keli Yang, Fangyan Yuan, Ting Gao, Zewen Liu, Chang Li, Rui Guo, Shaobo Xiao, Yongxiang Tian, Danna Zhou

**Affiliations:** 1 Key Laboratory of Prevention and Control Agents for Animal Bacteriosis (Ministry of Agriculture and Rural Affairs), Hubei Provincial Key Laboratory of Animal Pathogenic Microbiology, Institute of Animal Husbandry and Veterinary, Hubei Academy of Agricultural Sciences, Wuhan 430070, China; liuwei@hbaas.com (W.L.); jpz3865012@163.com (P.J.); keliy6@126.com (K.Y.); fyyuan@hbaas.com (F.Y.); gaoting2017@hbaas.com (T.G.); liuzwen2004@hbaas.com (Z.L.); lichang1113@hbaas.com (C.L.); hlguorui@163.com (R.G.); 2 Hebei Key Laboratory of Preventive Veterinary Medicine, College of Animal Science and Technology, Hebei Normal University of Science and Technology, Qinhuangdao 066004, China; songtaoer@126.com; 3 State Key Laboratory of Agricultural Microbiology, Key Laboratory of Preventive Veterinary Medicine of Hubei Province, Division of Animal Infectious Diseases, College of Veterinary Medicine, Huazhong Agricultural University, Wuhan 430070, China; vet@mail.hzau.edu.cn

**Keywords:** *Mycoplasma hyopneumoniae*, multi-antigen, chimeric vaccine, immune responses

## Abstract

*Mycoplasma hyopneumoniae* is the etiological agent of porcine enzootic pneumonia (EP), leading to a mild and chronic pneumonia in swine. Relative control has been attained through active vaccination programs, but porcine enzootic pneumonia remains a significant economic challenge in the swine industry. Cellular immunity plays a key role in the prevention and control of porcine enzootic pneumonia. Therefore, the development of a more efficient vaccine that confers a strong immunity against *M. hyopneumoniae* is necessary. In this study, a multi-antigen chimera (L9m6) was constructed by combining the heat-labile enterotoxin B subunit (LTB) with three antigens of *M. hyopneumoniae* (P97R1, mhp390, and P46), and its immunogenic and antigenic properties were assessed in a murine model. In addition, we compared the effect of individual administration and multiple-fusion of these antigens. The chimeric multi-fusion vaccine induced significant cellular immune responses and high production of IgG and IgM antibodies against *M. hyopneumoniae*. Collectively, our data suggested that rL9m6 chimera exhibits potential as a viable vaccine candidate for the prevention and control of porcine enzootic pneumonia.

## 1. Introduction

*Mycoplasma hyopneumoniae* is the causative agent of porcine enzootic pneumonia (EP), a chronic respiratory disease that affects swine [1]. This highly contagious organism has a worldwide distribution [2]. A study conducted in 125 farrow-to-finish pig herds demonstrated that *M. hyopneumoniae* is the major pathogen responsible for pneumonia-like gross lesions, with a detection positive rate of 70.8% among pigs over 22 weeks old [3]. The initial mycoplasmal infection frequently becomes complicated by secondary viral and bacterial infections [4], leading to the development of more severe pulmonary lesions and greater production losses. Saade et al. [5] assessed the severity of the clinical signs and the development of various coinfections or superinfections; the results indicated that *M. hyopneumoniae* frequently co-infected with other viruses or bacteria to exacerbate associated disease symptoms. EP prophylaxis entails the administration of antibiotics, implementation of management protocols, and vaccination, which is deemed the most effective control measure [6]. Vaccination with attenuated live and inactivated adjuvanted vaccines is frequently used worldwide to control *M. hyopneumoniae* infections [7]. However, the production costs of these vaccines are expensive, mainly attributed to the fastidious in vitro growth requirements of this microorganism [8]. Moreover, the efficacy of the cellular immune response induced by these vaccines may be limited. Therefore, it is necessary to develop more efficient and cost-effective vaccines against EP.

Significant endeavors have been made to enhance vaccine efficacy through the implementation of various strategies [9,10,11]. Subunit vaccines are proven to be effective and are widely used in the prevention and control of human papillomavirus (Cervarix, GlaxoSmithKline, Brentford, UK) [12], porcine circovirus 2 (Ingelvac CircoFLEX, Boehringer Ingelheim, Ingelheim am Rhein, Germany) [13], and piglet *Escherichia coli* (IDT Biologika GmbH, Dessau-Roßlau, Germany), et al. The reverse vaccinology approach has identified several secreted or surface proteins of *M. hyopneumoniae* that have the potential to be included in subunit vaccines [14,15]. Some of these antigen candidates include P97 adhesin and its C-terminal region (P97R1), as well as 46 kDa surface antigen (P46). Genetically engineered vaccines composed of a single recombinant antigen often exhibit low immunogenicity [16]. Therefore, it is imperative to enhance their immunogenic potential by means of combination strategies. Our previous research has demonstrated that the membrane-associated lipoprotein mhp390 (68 kDa) can specifically bind to cilia, and mediates adhesion and colonization of *M. hyopneumoniae*. Additionally, this protein induces significant apoptosis in lymphocytes and monocytes derived from peripheral blood mononuclear cells (PBMCs), as well as primary porcine alveolar macrophages (PAMs) [17]. The antibody against mhp390 effectively inhibited *M. hyopneumoniae*-induced apoptosis and the adhesion of *M. hyopneumoniae* to PAM cells [17,18].

The aims of the present study were: (i) to generate a recombinant chimeric protein comprising the P97R1, mhp390, and P46 antigens of *M. hyopneumoniae*, (ii) to assess the capacity of this chimeric protein to elicit both humoral and cellular immune responses in mice, (iii) to compare the immune response elicited by rL9m6 versus the commercial vaccine, and (iv) to compare the immune response elicited by individual administration versus multiple fusion of these antigens.

## 2. Materials and Methods

### 2.1. Bacterial Strains, Cultivation Conditions, and Genomic DNA Extraction

*Escherichia coli* strains DH5a and BL21 (DE3) were cultured in a Luria Bertani (LB) medium supplemented with kanamycin (50 mg/mL) or ampicillin (100 mg/mL), and incubated at 37 °C and 200 rpm. The *M. hyopneumoniae* strain 168, which was isolated from the no. 168 Er-hua-nian pig in 1974, demonstrated the typical clinical and pathogenic characteristics of mycoplasmal pneumonia of swine [19]. The strain 168 was cultured in a KM2 cell-free medium at 37 °C, followed by harvesting of the mycoplasma culture through centrifugation at 12,000× *g* for 30 min. Subsequently, total genomic DNA was extracted from mycoplasma cultures using a TIANamp Bacteria DNA Kit (Tiangen, Beijing, China) in accordance with the manufacturer’s instructions.

### 2.2. Primers, Amplification of Coding Sequences, and Site-Directed Mutagenesis

SignalP (http://www.cbs.dtu.dk/services/SignalP/ accessed on 16 January 2018) was utilized for the prediction of potential signal peptides, while TMHMM (http://www.cbs.dtu.dk/services/TMHMM/ accessed on 16 January 2018) was employed to detect transmembrane domains. The primers utilized in the current study are listed in Table 1, with a linker sequence (GGCAGCGGCAGCGGCAGCGGCAGC) appended to the forward primers. In brief, a synthetic chimeric gene encoding the R1 repeat region of P97, the membrane-associated lipoprotein gene mhp390, and the surface antigen gene P46 of *M. hyopneumoniae* were successfully cloned in our laboratory. P97R1, mhp390, and P46 genes, which harbor TGA codons in their nucleotide sequences, were subjected to site-directed mutagenesis. The mutations obtained were validated through DNA sequencing using the ABI3730 capillary sequencer.

### 2.3. Expression, Characterization, and Purification of Recombinant Proteins

Recombinant plasmids, including pET30a-P97R1, pET30a-mhp390, pET30a-P46, and pET30a-LTB-linker-P97R1-linker-mhp390-linker-P46 were constructed for transformation into *E. coli* BL21 (DE3). The 6× His-tagged recombinant proteins (P97R1, mhp390, P46, and LTB-P97R1-mhp390-P46) were expressed and subsequently purified using Ni Sepharose 6 Fast Flow (Cytiva, Marlborough, MA, USA). The purified recombinant proteins were separated by 12% SDS-PAGE gels, and their concentration was quantified through the BCA Protein Assay Kit (Beyotime, Shanghai, China). The proteins were then stored at −70 °C for future use.

### 2.4. Experimental Design and Immunization Routes

All animal experiments were conducted in compliance with the International Guiding Principles for Biomedical Research Involving Animals (1985). The animal study was approved by the Ethics Committee of the Institute of Animal Husbandry and Veterinary, Hubei Academy of Agricultural Sciences (Wuhan, China; identification code: SCXK(E)2020–0018, date of approval: 31 March 2022). Seven groups, each consisting of six female BALB/c mice (Hubei Experimental Animal Centre, Wuhan, China), were randomly assigned and intramuscularly (IM) inoculated on day zero as follows (Table 2): group 1 received 100 μL of phosphate-buffered saline (PBS); group 2 received 100 μL of phosphate-buffered saline (PBS) with 10% Montanide™ Gel02 PR vaccine adjuvant as a negative control; group 3 received rP46; group 4 received rP97R1; group 5 received rMhp390; group 6 received the chimeric protein LTB-P97R1-mhp390-P46 (rL9m6); and group 7 was administered with 100 μL Ingelvac MycoFLEX^®^ commercial vaccine (Boehringer-Ingelheim, Ingelheim am Rhein, Germany) as a positive control. Animals from groups 3, 4, 5, and 6 were immunized with a dose of 50 μg for each protein with 10% Montanide™ Gel02 PR vaccine adjuvant (a water-based adjuvant manufactured by Seppic, France). Each animal received an identical dose and formulation 35 days after the first inoculation. Blood samples were obtained from the tail of mice at three time points: 35, 42, and 56 days postinoculation (DPI). The sera were processed and stored at −80 °C until testing. On day 56, the mice were humanely euthanized, and their infected tissues were collected aseptically for culture and histopathological analysis.

### 2.5. Assessment of the Humoral Immune Response via Indirect ELISA Utilizing M. hyopneumoniae Extract

The IgG antibodies generated by immunization with each vaccine formulation were assessed via ELISA using the crude extract of *M. hyopneumoniae* 168, as described by Simionatto et al. [20]. Microtiter plates were coated with 400 ng/well of the crude extract of Mhp168 in 50 mM carbonate-bicarbonate buffer (pH 9.8) at 4 °C overnight. The plates were washed with PBS-T and subsequently incubated with a blocking buffer consisting of non-fat dry milk at 5% concentration, maintained at a temperature of 37 °C for a duration of 2 h. After washing with PBS-T, the wells were exposed to mouse serum diluted in a ratio of 1:800 in blocking buffer for another 2 h at 37 °C. After further washing with PBS-T, HRP-conjugated affinipure goat anti-mouse IgG peroxidase conjugate (Proteintech, Rosemont, IL, USA) was added to the wells and allowed to incubate at a temperature of 37 °C for 1 h. After PBS-T washing, the reactions were developed using TMB (Biodragon, Beijing, China). The color reaction was allowed to develop for 15 min after PBS-T washing and stopped with 100 μL of 2 M H_2_SO_4_. Absorbance at 450 nm was determined with a microplate reader. Triplicate samples were used to calculate mean and standard deviation (S.D.) values.

The IgM antibody elicited by immunization with each vaccine formulation was evaluated via ELISA using the crude extract of *M. hyopneumoniae* 168, following a procedure similar to that for IgG detection. The wells were incubated with mouse serum diluted in a ratio of 1:800 in blocking buffer at 37 °C for 2 h. A 1:5000 dilution of HRP-conjugated affinipure goat anti-mouse IgG peroxidase conjugate (Proteintech, Rosemont, IL, USA) was used as the secondary antibody. The absorbance was measured at 450 nm using a microplate reader.

### 2.6. Quantification of IFN-γ Expression via ELISA

The mouse splenocytes (1 × 10^6^ cells/mL) were cultured in 24-well plates using RPMI 1640 medium (Thermo Fisher, Wilmington, Massachusetts, USA), supplemented with 10% fetal bovine serum and incubated at 37 °C in a humidified incubator containing 5% CO_2_; cells were stimulated with *M. hyopneumoniae* 168 at a concentration of 10 μg/mL. The supernatant was harvested 72 h post-stimulation and clarified by centrifugation to remove cellular debris. The presence of IFN-γ was evaluated with mouse IFN-γ ELISA kits (Beyotime Biotechnology, Shanghai, China) in accordance with the instructions provided by the manufacturer. The concentrations of IFN-γ in the samples were quantified through standard curves.

### 2.7. Intracellular Cytokine Staining and Flow Cytometry Analysis

The antigen-specific CD4^+^ and CD8^+^ T-lymphocyte responses to each immunized group were measured through an intracellular cytokine staining (ICS) assay. Briefly, 1 × 10^6^ splenocytes were seeded in a 6-well plate and treated with *M. hyopneumoniae* protein at a concentration of 10 μg/mL. Cells were labeled with APC anti-mouse CD3 antibody (Biolegend, San Diego, CA, USA), PE anti-mouse CD4 antibody (Biolegend), and FITC anti-mouse CD8a antibody (Biolegend) (PBS:CD3:CD4:CD8a = 377:10:5:8) at 4 °C for 25 min, away from light. After being washed twice with cold PBS buffer, cells were resuspended in PBS and analyzed using Accuri C6 Plus Flow Cytometry (BD Biosciences, Franklin Lakes, NJ, USA).

### 2.8. Statistical Analysis

The statistical analysis was conducted using the student’s *t*-test to evaluate potential differences in humoral and cellular immune responses among various groups. The data are presented as mean ± standard deviation (SD). A *p*-value < 0.05 was considered significant, while a *p*-value < 0.01 was considered highly significant.

## 3. Results

### 3.1. Amplification of Target Genes and Purification of Recombinant Proteins

Three genes encoding the immunodominant proteins, namely the R1 region of P97 (MHP168_110), surface protein mhp390 (MHP168_418), and P46 surface antigen (MHP168_522), were selected for cloning and expression in *E. coli*. The possible cleavage site of the signal peptide in P46 was positioned between pos. 37 and 38 amino acids, and the transmembrane domain spanned residues 9aa to 31aa. Thus, we cloned the coding region of P46 without the transmembrane domain. The putative signal peptide cleavage site of mhp390 was predicted to be located between residues 26aa and 27aa, while a possible transmembrane helix was identified in the N-terminal region spanning residues 9 to 25. The coding region of mhp390, excluding the signal peptide sequence, was selected for cloning.

**Figure 1:**
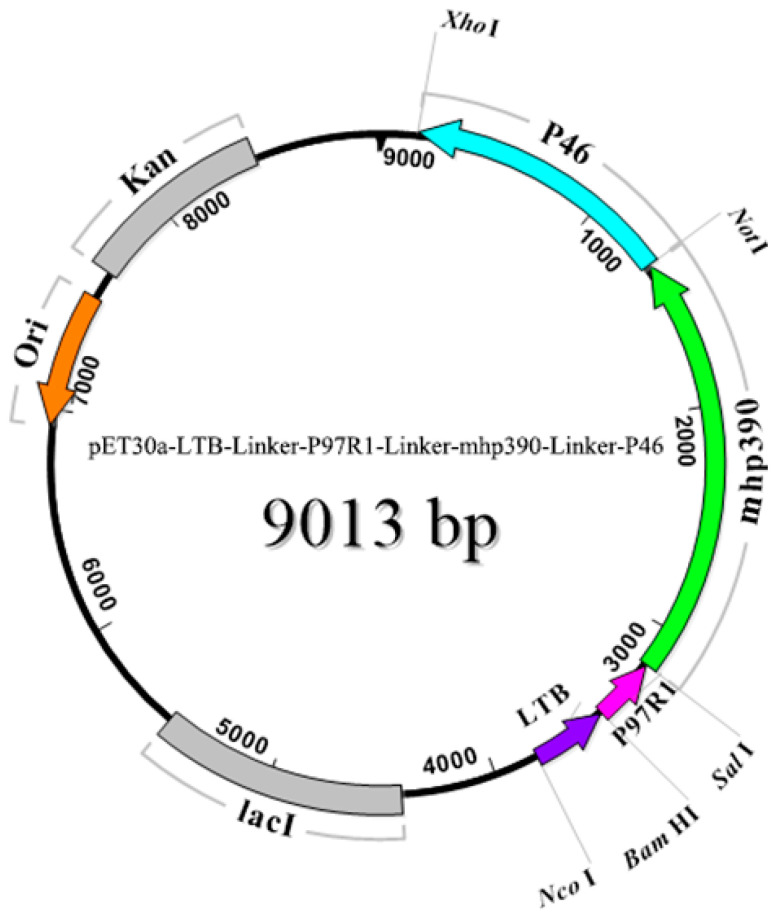
Schematic representation for the construction of rL9m6 chimera. The P46, mhp390, and R1 repeat region of P97 was amplified from Mhp168 genomic DNA. The recombinant L9m6 corresponds to the pET-30a vector backbone. The P46 gene was inserted between the *Not* I and *Xho* I site, and then the mhp390 gene was cloned into *Sal* I and *Not* I site. The P97R1 was cloned into *Bam*H I and *Sal* I site, while the LTB was amplified from the genomic DNA of *E. coli* and cloned into *Nco* I and *Bam*H I site.

The TGA codon in mycoplasma species is utilized as a tryptophan amino acid instead of serving as a translational stop [21]. To produce integral proteins in *E. coli*, substitution of TGA with the universally recognized tryptophan codon TGG is necessary [1,22]. Therefore, we employed in vitro mutagenesis techniques to replace the bases, and confirmed the correct base changes through DNA sequencing analysis. All amplicons were successfully cloned and characterized using restriction enzymes, demonstrating the expected fragment sizes. The schematic construction and restriction enzyme sites utilized in the creation of the L9m6 chimeric plasmid are depicted in Figure 1. A flexible linker sequence was incorporated between each fusion gene to enable the independent movement of protein domains while preserving their distinct functions.

**Figure 2:**
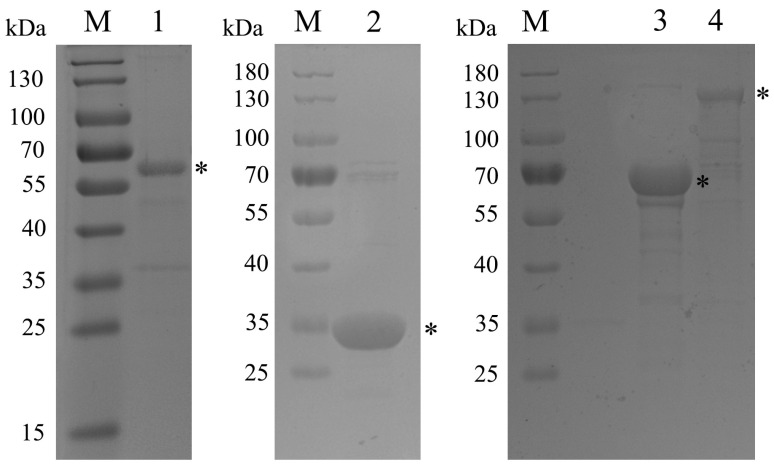
SDS-PAGE analysis of purified recombinant protein. M, Protein Marker (kDa); lane 1, purified rP46 by Ni-NTA column; lane 2, purified rP97R1 by Ni-NTA column; lane 3, purified rMhp390 by Ni-NTA column; lane 4, purified rL9m6 by Ni-NTA column. * indicate the individual recombinant protein.

The recombinant proteins were analyzed by protein electrophoresis, which revealed distinct bands with apparent molecular masses (Figure 2). Furthermore, all expressed recombinant proteins were purified using the AKTA FPLC (Amersham Biosciences UPC-900, Piscataway, NJ, USA) system and found to be soluble.

### 3.2. Cellular Immune Responses to the rL9m6 Fusion Protein Formulated with Gel02 PR Adjuvant

**Figure 3:**
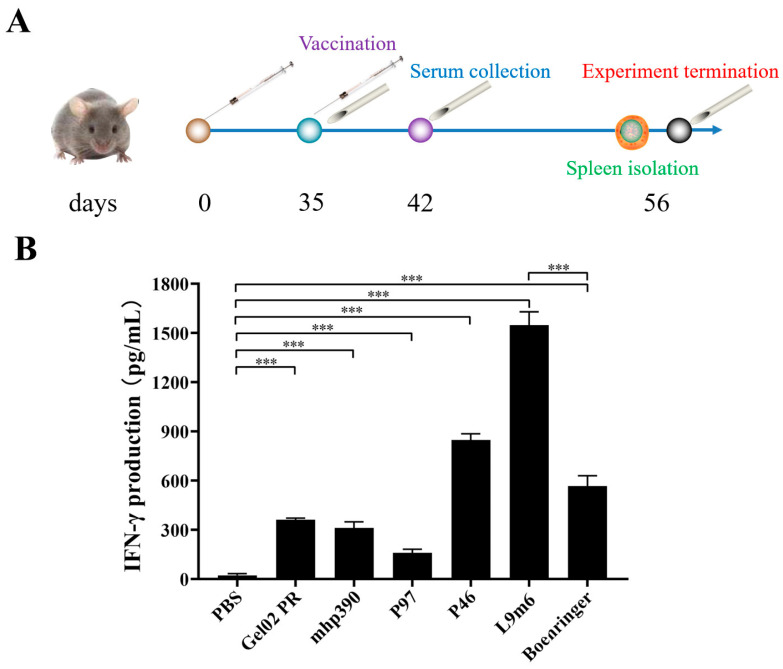
Study design and IFN-γ production from each vaccinated group. (**A**) Overview of experimental design. Forty-two female BALB/c mice were randomly allocated to 7 groups. Each animal was immunized 35 days post-primary inoculation, according to Table 2. Blood samples were obtained from the tail of mice at three time points. (**B**) The production of IFN-γ in the supernatant of splenocytes harvested from immunized mice after in vitro restimulation. Splenocytes were isolated 3 weeks after secondary immunization and stimulated with 10 μg/mL extract protein of *M. hyopneumoniae* for 72 h. The presence of IFN-**γ** was evaluated using mouse IFN-**γ** ELISA kits (Beyotime Biotechnology, Shanghai, China) in accordance with the manufacturer’s instructions. The concentrations of IFN-**γ** were quantified using standard curves. The presented values represent means ± standard errors (SE) from three independent experiments. *** *p* < 0.001 indicate statistically significant difference among various groups.

**Figure 4:**
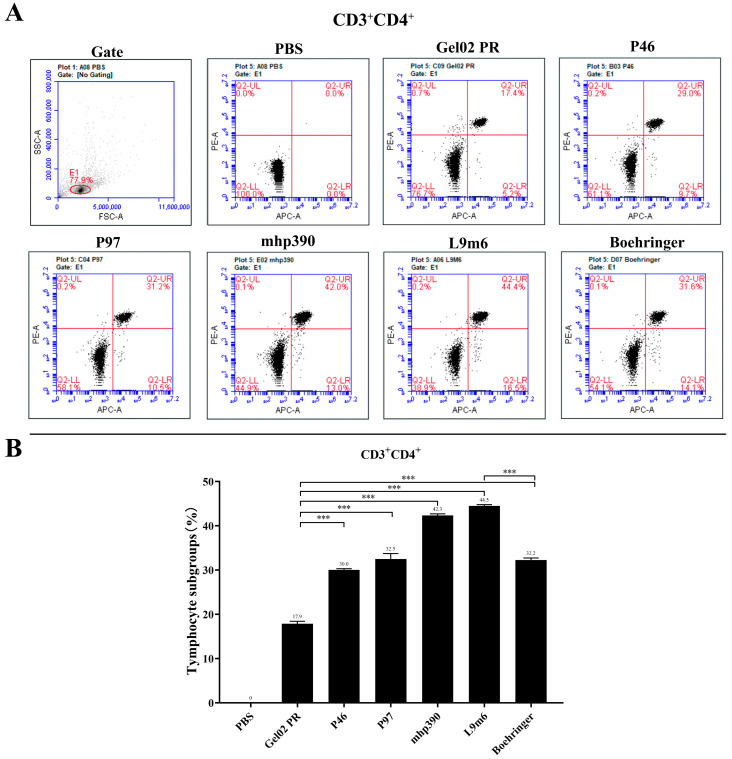
Proportion of splenic CD4^+^ T cells measured by intracellular cytokine staining. (**A**) The count of CD3^+^, CD4^+^, and CD8^+^ T lymphocytes in the spleen was measured by flow cytometry. Representative flow cytometric dot plot demonstrates the gating strategy employed for analyzing subsets of T lymphocytes based on forward and sidelight scatter. Dot plots exhibiting double-positive staining indicate the CD4^+^ T cells (Q-UR). (**B**) The CD4^+^ T cells rates in the spleen, shown as a percentage, were assessed by flow cytometry. *** *p* < 0.001 indicate statistically significant difference among various groups.

**Figure 5:**
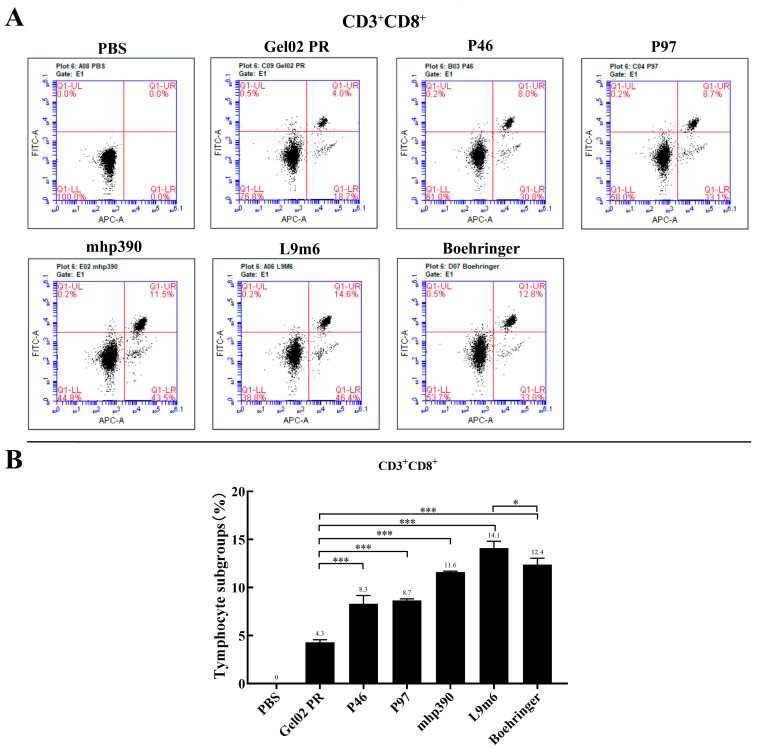
Proportion of splenic CD8^+^ T cells measured by intracellular cytokine staining. (**A**) The count of CD3^+^, CD4^+^, and CD8^+^ T lymphocytes in the spleen was quantified by flow cytometry. Representative flow cytometric dot plot demonstrates the gating strategy employed for analyzing subsets of T lymphocytes based on forward and sidelight scatter. Dot plots exhibiting double-positive staining indicate the CD8^+^ T cells (Q-UR). (**B**) The CD8^+^ T cells rates in the spleen, shown as a percentage, were assessed by flow cytometry. * *p* < 0.05, and *** *p* < 0.001 indicate statistically significant differences among various groups.

IFN is a crucial cytokine involved in Th1-type cellular immune responses [23]. Cellular immune responses were assessed post-booster immunization by measuring the IFN-γ expression in splenocytes that had been stimulated with *M. hyopneumoniae* protein in vitro. Figure 3 demonstrates a significant increase in IFN-γ production from the single protein vaccinated group (*p* < 0.001) compared to the PBS group. Notably, the rL9m6 elicited the highest level of IFN-γ expression out of all vaccinated groups. The expression level of IFN-γ induced by rL9m6 was significantly elevated, about 2.73-fold, compared to that induced by the commercial vaccine (*p* < 0.001). Moreover, the induction of IFN-γ expression by rL9m6 was significantly higher than that achieved through single protein immunization (*p* < 0.001). The counts of CD3^+^, CD4^+^, and CD8^+^ T lymphocytes in the spleen were quantified using flow cytometry. The rL9m6-immunized group exhibited significantly higher levels of both CD3 ^+^ CD4^+^ and CD3^+^ CD8^+^ T lymphocytes compared with the single protein immunized group. Among the groups immunized with single proteins, mice vaccinated with mhp390 exhibited the highest percentages of both CD4^+^ and CD8^+^ T lymphocytes. As presented in Figure 4 and Figure 5, the commercial vaccine also induced high percentages of CD4^+^ T lymphocytes, albeit slightly lower than that for rL9m6-immunized group. The percentage of CD4^+^ T lymphocytes induced by rL9m6 was 1.38-fold elevated when compared to the commercial vaccine (*p* < 0.001).

### 3.3. Humoral Immune Response to the rL9m6 Fusion Protein Formulated with Gel02 PR Adjuvant

**Figure 6:**
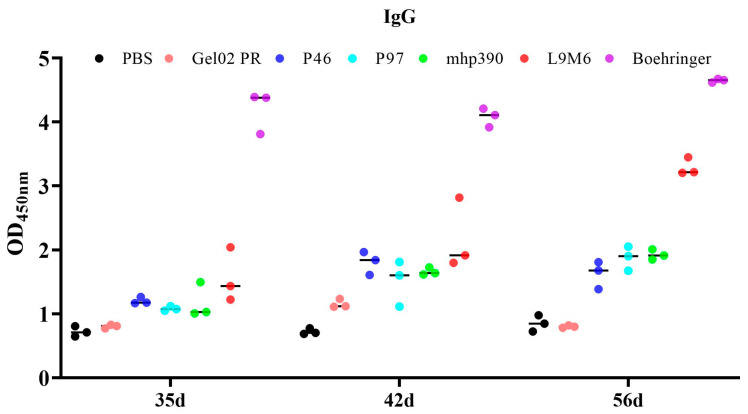
The IgG antibody response in mice immunized with each vaccine formulation. Serum samples were collected 5, 6, and 8 weeks after primary immunization. The IgG level of seroconversion induced by each vaccinated group was determined via indirect ELISA utilizing *M. hyopneumoniae* whole cell extract.

The IgG antibody response induced by each vaccinated group was determined via indirect ELISA using the whole cell extract of *M. hyopneumoniae* after intramuscular immunization of BALB/c mice (Figure 6). Animals administered with the commercial vaccine exhibited a robust production of antibodies against native proteins of *M. hyopneumoniae*. All recombinant proteins induced a higher antibody response at 35 DPI compared to the negative control (PBS) or adjuvant-immunized mice. This response was constant until 56 DAI. Among the vaccines immunized groups, the commercial vaccine elicited the highest level of IgG antibodies, followed by the rL9m6 vaccine. The seroconversion induced by the rL9m6 vaccine was statistically equivalent to that induced by the single protein vaccine at 35 DPI and 42 DPI. However, at 56 DPI the rL9m6 vaccine elicited a significantly higher level of IgG antibodies compared to the single protein vaccine (*p* < 0.001).

**Figure 7:**
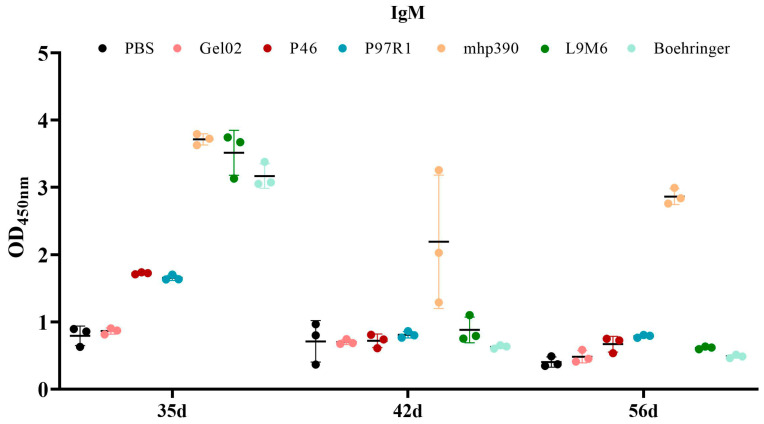
The IgM antibody response in mice immunized with each vaccine formulation. Serum samples were collected 5, 6, and 8 weeks after primary immunization. The IgM level of seroconversion induced by each vaccinated group was measured by indirect ELISA using the whole cell extract of *M. hyopneumoniae*.

The IgM antibody response elicited by each vaccinated group was assessed through indirect ELISA using the whole cell extract of *M. hyopneumoniae* (Figure 7). All vaccinated groups demonstrated a heightened IgM antibody response at 35 DPI, which declined at both 42 and 56 DPI. The rL9m6 vaccine induced a superior level of IgM compared to the commercial vaccine. Animals inoculated with rMhp390 produced the highest levels of IgM antibodies against *M. hyopneumoniae* native proteins, and these high levels of IgM persisted at 56 DPI.

## 4. Discussion

The development of recombinant subunit vaccines, comprising highly effective antigen compositions generated through diverse expression systems, has garnered increasing attention in recent years [24,25]. Subunit vaccines are considered safer as they do not contain live pathogens and lack the ability to replicate in the organism [16]. The production costs of *M. hyopneumoniae* vaccines are expensive, mainly attributed to the fastidious growth requirements of this microorganism in vitro [8]. The utilization of subunit vaccines for *M. hyopneumoniae* can result in reduced vaccine production costs when compared to inactivated vaccines. Usually, the protective capacity of a single antigen protein is constrained. Research has demonstrated that utilizing a combination of recombinant antigens is more advantageous in the development of vaccines [26]. Moreover, another issue arises when subunit vaccines are employed for immunizing large animals, such as cattle, as this leads to a significant increase in the quantity and cost of the subunit vaccines required, particularly when multiple antigenic proteins need to be prepared. To tackle this issue, the fusion of multiple recombinant antigens into a single fusion protein might simplify the vaccine preparation process and reduce the cost. In this study a fusion protein, containing LTB and three *M. hyopneumoniae* antigens, was constructed and evaluated for its capacity to confer immune protection in a murine model. The assessment of *M. hyopneumoniae* proteins’ antigenicity and immunogenicity in mice is a crucial step toward identifying antigens with potential to enhance the efficacy of the vaccines to be tested in pigs [6].

Membrane-associated lipoprotein mhp390 plays an important role in the adhesion of *M. hyopneumoniae* to swine tracheal cilia [17]. The mhp390 antibody could effectively block the binding of *M. hyopneumoniae* to PAM cells. In addition, mhp390 was able to induce significant apoptosis of PBMCs and PAMs, while its antibody could block the apoptosis progress [18]. The 46-kDa surface antigen (P46) has been tested as a DNA and subunit vaccine in mice. The results demonstrated that P46 induced high levels of IgG antibodies and IFN-γ expression when used as a DNA and subunit vaccine [20,23]. Attachment of *M. hyopneumoniae* to the respiratory epithelium is a crucial step in host colonization and is primarily facilitated by the membrane protein P97 [27,28]. This protein is located on the surface of outer membrane, and its role in adherence has been firmly established. The R1 region, located near the C-terminus of P97, is believed to be responsible for mediating adherence to swine cilia [29]. A minimum of eight tandem copies of the pentapeptide sequence (AAKPV/E) in R1 are required for binding to cilia, and antibodies stimulated by P97R1 may partially inhibit *M. hyopneumoniae*’s adherence to receptors on epithelial cell cilia [29]. Therefore, the primary strategy was to develop a chimeric vaccine that incorporates three immunogenic antigen determinants of *M. hyopneumoniae*, such as P97R1, mhp390, and P46, to increase the immunogenicity of the subunit vaccine.

In the present study, we propose the utilization of water-based adjuvants (Montanide™ Gel02 PR vaccine adjuvant) in subunit vaccines due to their ability to elicit specific humoral and cellular immune responses. This adjuvant has been extensively employed in vaccine research to prevent diseases in porcine, avian, and fish species [30,31,32]. It offers a substantial improvement in immune response while maintaining an equivalent safety profile to aluminum adjuvant [33].

The heat-labile enterotoxin B subunit (LTB) has been extensively investigated as a potent vaccine adjuvant. LTB was utilized as an adjuvant conjugated to rabies virus-like particles (VLP), resulting in significantly enhanced humoral and cellular immune responses specific to the rabies virus in dog and mouse models [34]. Moreover, a rotavirus VLP vaccine containing purified recombinant LTB was administered to mice models. The findings indicated that intrarectal vaccination with LTB served as a potent adjuvant for B cell responses and induced a specific Th17 T cell response to the rotavirus VLP [35]. Recent research suggests that the adjuvanticity of LTB is mediated by enhancing dendritic cell turnover in the spleen and increasing their capacity to function as antigen-presenting cells when encountering T cells [36]. LTB also mediates the clustering of B and T cells, as well as the delay or arrest of T cell division after endocytosis or B-cell receptor (BCR) uptake of antigens in a ganglioside-dependent manner [37].

The cellular and humoral immune responses elicited by the chimeric subunit vaccine were assessed following intramuscular inoculation in BALB/c mice. The results demonstrate that the chimeric vaccine can elicit robust humoral immune responses, which are statistically superior to those induced by most individual proteins administered separately. In most assays, the fusion protein exhibited superior efficiency. Our data are consistent with previous studies that underscore the advantages and performance of chimeric proteins for application in vaccine development [38,39]. However, the IgG antibodies elicited by the chimeric vaccines fell short of expectations, with IgG levels lower than those observed in commercial vaccines (Ingelvac MycoFLEX, Ingelheim am Rhein, Germany). The IgM antibody response was elevated at 35 DPI, but decreased at both 42 and 56 DPI. The rL9m6 vaccine induced a superior level of IgM compared to the commercial vaccine.

In addition to stimulating humoral responses, the induction of cellular immunity is also critical for providing protection [40]. It is noted that the commercial vaccine was observed to elicit a high level of IgG and IgM antibodies in mice that could recognize native proteins of *M. hyopneumoniae*, while failing to stimulate IFN-γ gene expression. The level of IFN-γ induced by rL9m6 was significantly elevated, about 2.73-fold, compared to that induced by the commercial vaccine. Significant differences in IFN-γ levels were observed between groups, when the cells were administered with chimeric vaccine and corresponding individual recombinant protein. The counts of CD3^+^, CD4^+^, and CD8^+^ T lymphocytes in the spleen were quantified using flow cytometry. As shown in Figure 4 and Figure 5, the commercial vaccine also induced high percentages of both CD4^+^ and CD8^+^ T lymphocytes, albeit lower than that for the rL9m6 immunized group. To a certain degree, high numbers of CD3 ^+^ CD4^+^ and CD3^+^ CD8^+^ cells tend to enhance the therapeutic efficacy against *M. hyopneumoniae* [41].

The effectiveness of vaccines against many infectious diseases is predominantly reliant on the presence of serum antibodies, as well as augmentation in cell-mediated and local immunity [42,43,44,45]. However, protection efficiency against porcine enzootic pneumonia was not found to be directly correlated with serum antibody concentrations [46]. In general, cell-mediated immune responses are considered more crucial for protection against Mycoplasma induced pneumonia [41,47]. Previous research has demonstrated that the mhp390 and P97R1 antibodies could effectively block adherence of *M. hyopneumoniae* to epithelial cell cilia, while the mhp390 antibody could block the binding of *M. hyopneumoniae* to PAM cells and the apoptosis progress induced by *M. hyopneumoniae*. However, it remains unclear whether the production of antibodies against the rL9m6 vaccine increases the likelihood of blocking adherence of *M. hyopneumoniae* to epithelial cell cilia and offers more adequate protection during infection. These data will facilitate the identification of potential targets for a subunit vaccine against EP. The administration of fusion chimeric protein resulted in an increase in serum antibody, IFN-γ production, and CD4^+^ and CD8^+^ T lymphocytes, indicating that this approach holds great promise for the development of an effective vaccine. However, further investigation through challenge experiments in pigs is warranted.

## 5. Conclusions

In this study, we developed a chimera vaccine by fusing multiple key *M. hyopneumoniae* antigens into a single fusion protein (rL9m6) and assessed its capacity to elicit both cellular and humoral immune responses in a murine model. The heat-labile enterotoxin B subunit (LTB) and Gel02 PR were employed as molecular and water-based adjuvants, respectively, to enhance vaccine efficacy by eliciting superior immune responses. Notably, vaccination with the rL9m6 chimeric vaccine elicited significantly stronger cellular immune responses (both CD4^+^ and CD8^+^ T lymphocytes) than the commercial vaccine. Furthermore, the level of IFN-γ induced by rL9m6 was significantly elevated, about 2.73-fold, compared to that induced by the commercial vaccine. The chimeric vaccine also increased the production of both IgG and IgM antibodies against *M. hyopneumoniae*. Taken together, our findings suggest that rL9m6 chimera exhibits potential as a viable vaccine candidate to prevent and control *M. hyopneumoniae* infection.

## Figures and Tables

**Table 1:** Primers used for amplify antigen genes from *M. hyopneumoniae* and site-directed mutation.

Gene	Genbank Accession	Primer	Primer Sequence	Restriction Enzyme
LTB	MF990202.1	LTB-F ^a^	TTT*CCATGG*CTATGGCTCCCCAGACTATTACA	*Nco* I
		LTB-R ^a^	TCT*GGATCC*CATACTGATTGCCG	*Bam*H I
P97R1	ADQ90328.1	P97-F ^a^	TTT*GGATCC***GGCAGCGGCAGCGGCAGCGGCAGC**ATGTTACCTCAGCCGCCAGCAGCT	*Bam*H I
		P97-R ^a^	TTT*GTCGAC*AGCCATTGGGAAATAGTCTTCTTTTGGTTTATTT	*Sal* I
mhp390	AGM22196.1	mhp390-F ^a^	TTT*GTCGAC***GGCAGCGGCAGCGGCAGCGGCAGC**ATGAATTTCAAAAAATATTTAAAAT	*Sal* I
		mhp390-R ^a^	TCT*GCGGCCGC*ATTTTGTTCATCAATTAGTTTTAGAATTTCTTGC	*Not* I
P46	ADQ90718.1	P46-F ^a^	TTT*GCGGCCGC*A**GGCAGCGGCAGCGGCAGCGGCAGC**ATGACTTCTGATTCTAAACCAC	*Not* I
		P46-R ^a^	TTT*CTCGAG*TTAGGCATCAGGATTATCAACATTAGCTTTTGT	*Xho* I
P97R1	ADQ90328.1	P1 ^b^	TTT*GGATCC*ATGTTACCTCAGCCGCC	*Bam*HI *Xho* I
		P2 ^b^	TTT*CTCGAG*TTAGCCATTGGGAAAT	
mhp390	AGM22196.1	P3 ^b^	TCT*GGATCC*ATGAATTTCAAAAAATATTTAAAAT	*Bam*HI *Sal* I
		P4 ^b^	TTT*GTCGAC*TTAATTTTGTTCATCAATTAGTTT	
P46	ADQ90718.1	P5 ^b^	TTT*GGATCC*ATGAAAAAAATGCTTAG	*Bam*HI *Xho* I
		P6 ^b^	TTT*CTCGAG*TTAGGCATCAGGATTAT	

F: forward primers; R: reverse primers. ^a^ primers designed for construct the recombinant chimera. ^b^ primers designed for construct the single recombinant protein. Nucleotides in italics and underline denote the restriction enzyme cutting site. Nucleotides in bold denote the flexible linker sequence.

**Table 2:** Groups of mice and vaccine preparations used in this study.

Group	Immunogen	Dose	Route
Group 1	Phosphate-buffered saline	100 μL	i.m.
Group 2	Gel02 PR	100 μL PBS with 10% Gel02 PR	i.m.
Group 3	rP46	50 μg + 10% Gel02 PR	i.m.
Group 4	rP97R1	50 μg + 10% Gel02 PR	i.m.
Group 5	rMhp390	50 μg + 10% Gel02 PR	i.m.
Group 6	rL9m6	50 μg + 10% Gel02 PR	i.m.
Group 7	Boehringer	100 μL	i.m.

rL9m6: the recombinant LTB-P97R1-mhp390-P46 protein. i.m. intramuscular injection.

## Data Availability

Not applicable.
